# Storage of neural histamine and histaminergic neurotransmission is VMAT2 dependent in the zebrafish

**DOI:** 10.1038/s41598-017-02981-w

**Published:** 2017-06-08

**Authors:** Henri A. J. Puttonen, Svetlana Semenova, Maria Sundvik, Pertti Panula

**Affiliations:** Neuroscience Center and Department of Anatomy, University of Helsinki, Helsinki, Finland

## Abstract

Monoaminergic neurotransmission is greatly dependent on the function of the vesicular monoamine transporter VMAT2, which is responsible for loading monoamines into secretory vesicles. The role of VMAT2 in histaminergic neurotransmission is poorly understood. We studied the structure and function of the histaminergic system in larval zebrafish following inhibition of VMAT2 function by reserpine. We found that reserpine treatment greatly reduced histamine immunoreactivity in neurons and an almost total disappearance of histamine-containing nerve fibers in the dorsal telencephalon and habenula, the most densely innervated targets of the hypothalamic histamine neurons. The reserpine treated larvae had an impaired histamine-dependent dark-induced flash response seen during the first second after onset of darkness, implying that function of the histaminergic network is VMAT2 dependent. Levels of histamine and other monoamines were decreased in reserpine treated animals. This study provides conclusive evidence of the relevance of VMAT2 in histaminergic neurotransmission, further implying that the storage and release mechanism of neural histamine is comparable to that of other monoamines. Our results also reveal potential new insights about the roles of monoaminergic neurotransmitters in the regulation of locomotion increase during adaptation to darkness.

## Introduction

The function of most monoamine neurotransmitters is dependent on the vesicular release made possible by the vesicular monoamine transporter 2 (VMAT2). Serotonin and dopamine are loaded into secretory vesicles by VMAT2, and inhibition of VMAT2 leads to rapid depletion of these neurotransmitters through metabolism by the enzyme monoamine oxidase (MAO)^1^. Since noradrenaline is synthesized from dopamine inside secretory vesicles^2^, VMAT2 inhibition also lowers noradrenaline levels^1^. VMAT2 inhibition by the indole alkaloid reserpine was previously used as a treatment for psychotic conditions^3^. Depression has often been reported as a side-effect of reserpine treatment, which was a key factor in the development of the monoamine depletion theory of depressive disorders and the reserpine treated animal model for depression, although the monoamine hypothesis has recently been challenged^4–6^. Taken together, these findings underline the significance of monoamine dependent neurotransmission in brain physiology.

The role of VMAT2 in histaminergic neurotransmission remains unclear. Like the other monoamines, histamine has been described as a substrate for VMAT2^7^. In gastric enterochromaffin-like (ECL) cells, histamine storage is controlled by reserpine^8^, and reserpine induces changes in vesicle morphology and subcellular distribution of histamine^9^. However, histamine is not known to be significantly metabolized by MAO, but is rather metabolized by histamine N-methyltransferase (HNMT) in the brain, followed by subsequent metabolism by MAO^10, 11^. In an explant culture from fetal rat hypothalamus, most histamine-immunoreactive neurons display VMAT2-immunoreactivity, with some neurons showing no VMAT2-immunoreactivity^12^. Studies on the effect of reserpine on brain histamine have yielded mixed results. In the cat and mouse a decrease in brain histamine has been reported^13, 14^, while another study in mice and experiments in the rat have shown histamine levels to be largely unchanged^10, 15^. Reserpine has also been shown to inhibit L-histidine induced increase in brain histamine in rats and mice^10, 15^. The interpretation of these previous studies is complicated by the presence of extraneural histamine in brain mast cells, which contributes to histamine content in some brain regions^16^. A recent study in mast cell deficient mutant rats revealed histamine levels to be unchanged, while the levels of its main metabolite tele-methylhistamine were decreased^10^. The same study also showed an increase in the activity of histidine decarboxylase (HDC), the rate-limiting enzyme for histamine biosynthesis. The zebrafish lacks extraneuronal histamine, and is thus an optimal model for the study of the neurochemistry of histamine^17^.

The effects of reserpine on all monoamines, in particular the histaminergic neurons, genes associated with monoamine system and associated motor behaviours in the zebrafish (*Danio rerio*) were addressed in this study. We hypothesized that if neural histamine storage is VMAT2 dependent, reserpine should cause a decrease in brain histamine levels and histamine immunoreactivity. Also, histamine-dependent brain functions should be impaired.

## Results

### Behavioural phenotype after reserpine treatment

**Figure 1:**
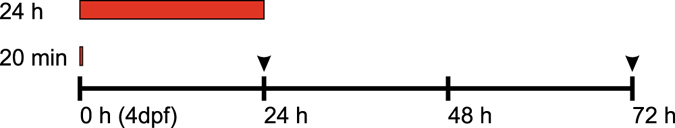
Reserpine treatment and experiment timeline. The reserpine treatment was started at 4 dpf and lasted either for 20 min (short treatment) or 24 h (long treatment). The treatment period is indicated by the red bar in the graph. Experiments were done at 24 h and 72 h after the start of the treatment, as indicated by the time points in the figure (arrows).

**Figure 2:**
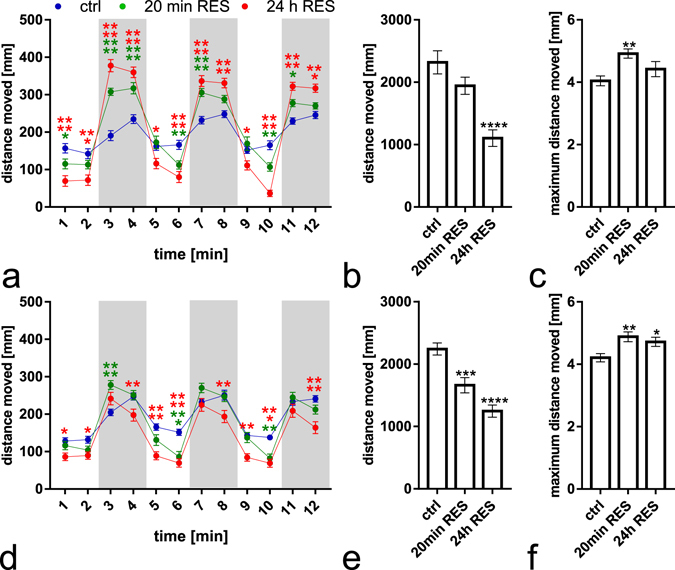
Behavioural profiles of reserpine treated larvae 24 h (**a**–**c**) and 72 h (**d**–**f**) after administration of reserpine under alternating light (2 min) and dark (2 min) periods. At all time points, the total distance moved was significantly decreased in reserpine treated larvae with both treatment durations manner during a 15 min trial (**b**,**e**). The maximum distance moved per bout during the same trial was increased in the 20 min treated group and mainly unaltered in the 24 h treated group (**c**,**f**). When altering between light and dark conditions (**a**,**d**, dark phase indicated with grey background), the reserpine treated larvae showed increased locomotion in the 2 min dark phase compared with controls at 24 h after administration of reserpine (**a**). When returning to the light phase, the locomotion activity decreased to the level preceding the dark phase. At 72 h after administration of reserpine (**d**), the difference between reserpine treated larvae and controls during the dark phase was less apparent, and the 24 h treated larvae actually move significantly less than controls at some time points. N = 48 in each group. Data presented as mean ± SEM. Statistical analyses were done using Kruskal-Wallis test followed by Dunn’s post hoc test (**b**,**c**) 1-way ANOVA (**e**,**f**) or 2-way ANOVA (**a**,**d**) followed by Tukey’s post hoc test. *p < 0.05, **p < 0.01, ***p < 0.001, ****p < 0.0001 according to the multiple comparison test.

Both the 20 min and 24 h reserpine treatments (see Fig. 1 for treatment setup) decreased the total distance moved observed during a 15 min tracking with lights on 24 h and 72 h after administration of reserpine (Fig. 2b, H(2) = 23.9 *p* < 0.0001 and e, F(2,141) = 22.07 *p* < 0.0001). The effect was dependent on the treatment duration, with the strongest locomotion depression observed in the 24 h treated group. No recovery of the behaviour was seen during the first three days after treatment. The maximum distance moved during a single bout of motion did not show any decrease, indicating that the motoneuron and muscular functions were intact (Fig. 2c and f). At some time points, the reserpine treated groups actually showed a significantly increased maximum distance moved (Fig. 2c, H(2) = 11.26 *p* = 0.0036 and f, F(2,141) = 5.719 *p* = 0.0041).

When the light was turned off for a 2 min dark phase, all fish reacted with an increased locomotor activity; a behavioural pattern that has been previously described for normal larvae^18^. At 24 h after administering reserpine, the increase in activity during the dark phase was much stronger in reserpine treated fish compared with controls (Fig. 2a, 2-way ANOVA interaction effect F(22,1551) = 24.91 *p* < 0.0001). At 72 h after treatment, the magnitude of the increase in locomotion activity observed was more similar across groups (Fig. 2d). After return to the light phase, the reserpine treated animals showed a similar hypoactivity in comparison to controls as before the dark phase.

**Figure 3:**
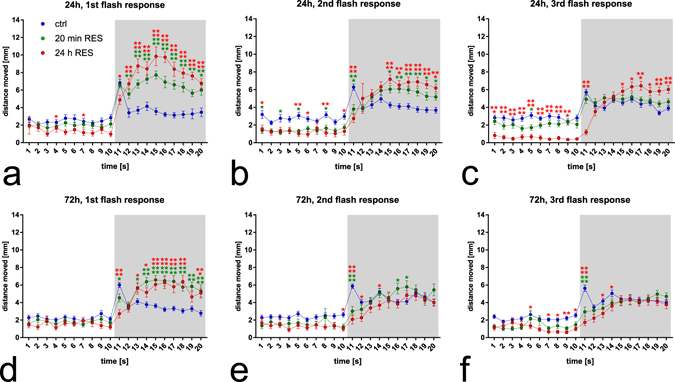
Locomotion during the light-dark transitions during the alternating light conditions trial from Fig. 1 at a 1 s resolution. Three consecutive transitions were analyzed at 24 h (**a**–**c**) and 72 h (**d**–**f**) after administration of reserpine. The control group shows a typical dark-induced flash response during the first second of darkness (time point 11). This activation was significantly decreased at all time points in the 24 h treated group. In the 20 min treated group, the decrease was seen at the 72 h time point, with the response being largely unaltered at the 24 h time point. The increase in locomotion during later in the dark phase could also be seen after the dark-flash time point, corresponding to results shown in Fig. 1. N = 48 in each group. Data presented as mean ± SEM. Statistical analyses were done using 2-way ANOVA followed by Tukey’s post hoc test. *p < 0.05, **p < 0.01, ***p < 0.001, ****p < 0.0001 according to the multiple comparison test.

Immediately after turning off the lights, the zebrafish demonstrate a dark-induced flash response, consisting of a sudden increase in locomotion activity during the first second of darkness by a histamine-dependent mechanism^18, 19^. We have previously shown this dark-induced flash response to be histamine dependent, as knock-down of histamine synthesis and inhibition of histamine receptor H1 mediated signaling abolish this response^19^. This dark-induced flash response was analyzed at each of the three light-dark transitions during the trial. 24 h after administration of reserpine the first dark-induced flash response was intact in both treatment groups, although its magnitude was significantly reduced in the group that was treated for 24 h (Fig. 3a, Tukey’s *p* = 0.0108 for the 24 h group). The second response was significantly weakened in both reserpine treated groups (Fig. 3b, Tukey’s *p* = 0.0003 and *p* < 0.0001 for 20 min and 24 h groups, respectively) and the third response was weakened in the groups treated for 24 h in comparison with non-treated control animals (Fig. 3c, Tukey’s *p* < 0.0001). At the 72 h time point, the first dark-induced flash was weakened in both reserpine treated groups when compared with control animals, and the second and third flash response was absent in both reserpine treated groups (Fig. 3d–f, Tukey’s *p* < 0.0001 except in 3d where *p* = 0.0339 for the group treated for 20 min). Although the dark-induced flash response was eliminated, the increased general activity during the dark phase was still present in the reserpine treated groups when compared with the non-treated control animals.

### Effect of reserpine on the morphology of histamine immunoreactivity in the zebrafish brain

**Figure 4:**
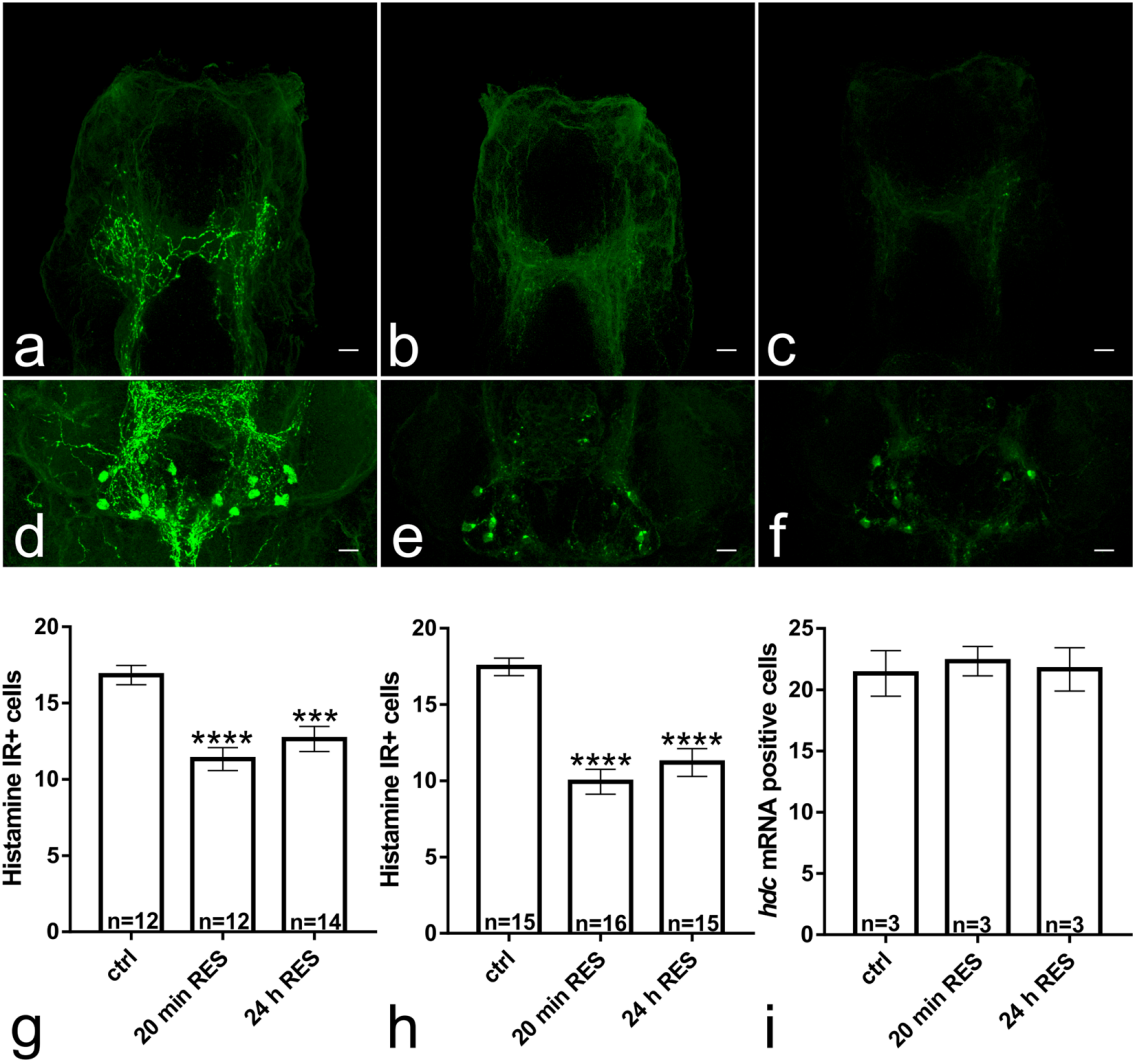
The larvae treated with 40 mg/L reserpine for 20 min (**b**) and 24 h (**c**) showed a clearly diminished number of histamine immunoreactive fibers in the telencephalon compared with controls (**a**) 72 h after administration of reserpine. A similar effect was seen in the hypothalamus, where larvae in the 20 min (**e**) and 24 h (**f**) treated groups had a clearly diminished number of histamine immunoreactive cells in comparison to controls (**d**). Some cell bodies showing clear immunoreactivity were still seen in both treatment groups. The dense network of hypothalamic histamine fibres (as seen in **d**) was also not present in the reserpine treated fish (**e**,**f**). The staining pattern observed was similar at 24 h after administration of reserpine (data not shown). In order to quantify the effect on the cell population, the number of cells showing clear cytoplasmic histamine immunoreactivity was counted at 24 h and 72 h after administration of reserpine (**g**,**h**, respectively). At all time points, the number of cells was significantly decreased by 30–40%. In order to verify that the reduction in histamine immunoreactivity was not due to destruction of histamine neurons, we counted the number of *hdc* mRNA positive cells in brains collected 72 h after administration of reserpine (**i**). No difference was seen between reserpine treated and control brains, indicating that the histamine neurons were intact. Statistical analyzes were done using 1-way ANOVA (**g**,**i**), followed by Tukey’s multiple comparison tests, respectively. Data are presented as mean ± SEM. ***p < 0.001, ****p < 0.0001 according to the multiple comparison test, in comparison with the control group. Scale bar 10 µm. The brains are in a horizontal orientation, with the anteroposterior axis pointing upwards.

Brains collected 72 h after start of reserpine treatment showed strongly diminished histamine fiber immunoreactivity in the telencephalon in comparison with controls (Fig. 4a–f). The number of histamine immunoreactive cells was also significantly decreased by approximately 40% at both time points after reserpine treatment when compared with control values (Fig. 4g and h, F(2,35) = 13.9 *p* < 0.0001 and F(2,43) = 26.34 *p* < 0.0001, respectively). Interestingly, the neurons that retained immunoreactivity showed a strongly immunoreactive soma but almost no immunoreactive projections that were abundant in the control brains (Fig. 4a–f).

### *hdc* mRNA colocalizes with *vmat2* mRNA in the brain

**Figure 5:**
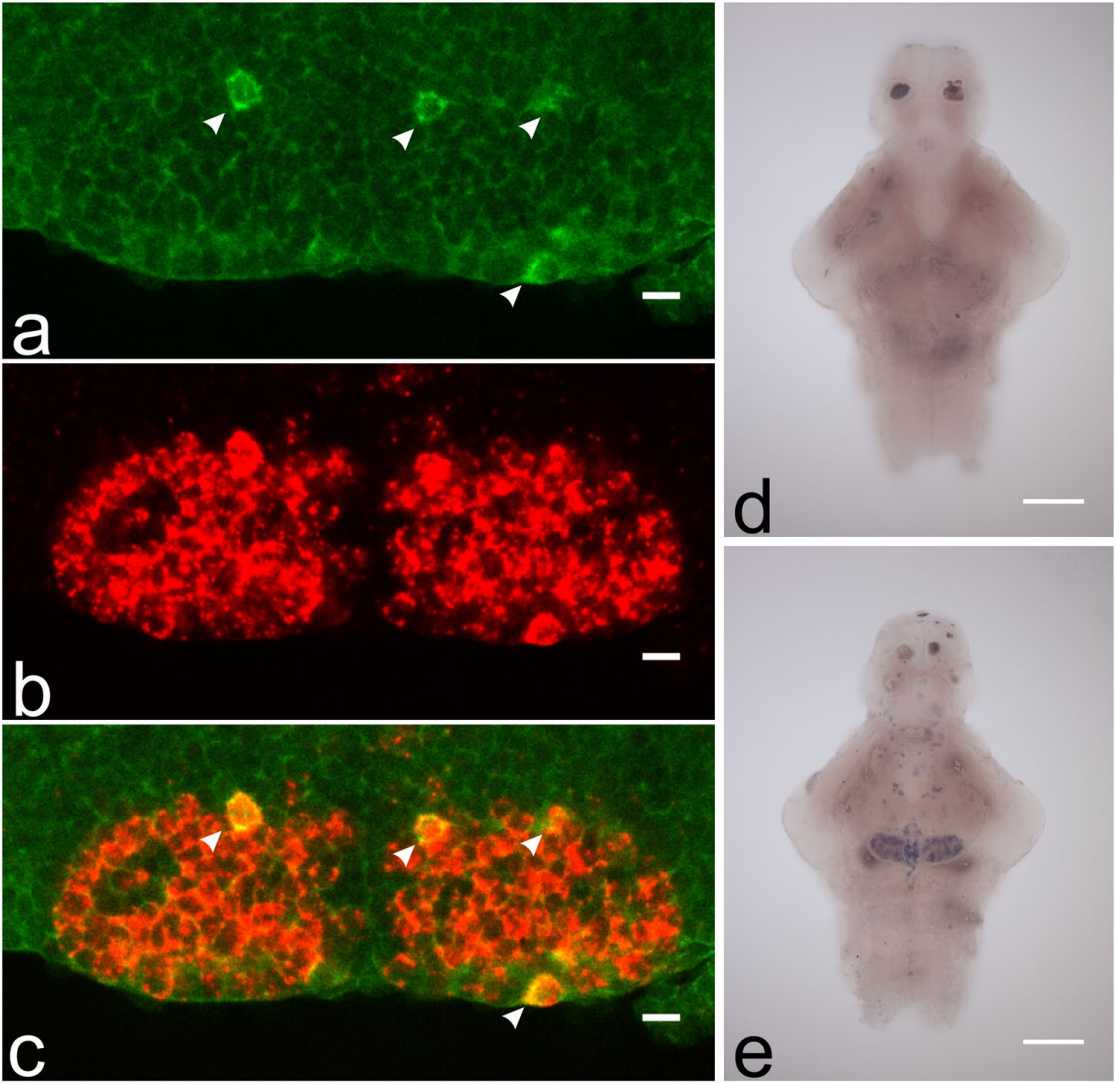
Expression of *vmat1*, *vmat2* and *hdc* in the hypothalamus. Virtually all *hdc* mRNA positive neurons seen (green, pointed out by white arrows) also express *vmat2* mRNA (red) (**a**–**c**). *n* = 6 brains, representative single optical sections from one sample are shown. Red signal is developed using TAMRA tyramide and green signal using DY-647P1 tyramide. We found no expression of *vmat1* in the zebrafish brain (**d**), although *vmat2* was present in multiple brain regions (**e**). Scale bar 10 µm for (**a**–**c**) and 100 µm for (**d**–**e**).

Double fluorescent *in situ* hybridization showed nearly complete coexpression of *hdc* and *vmat2*, indicating that *vmat2* is indeed present in most if not all histaminergic neurons (Fig. 5a–c). In addition, we saw no change in the number of *hdc* mRNA positive neurons after reserpine treatment (Fig. 4i), suggesting that the decreased histamine immunoreactivity is indeed due to depletion of histamine and not the destruction of the histaminergic neurons. We also investigated the possibility of *vmat1* expression in the larval zebrafish brain using sensitive chromogenic *in situ* hybridization. However, we saw no expression of *vmat1* transcript in the brain, while *vmat2* was strongly expressed in different brain areas (Fig. 5d,e).

### Genes regulated by reserpine administration

**Figure 6:**
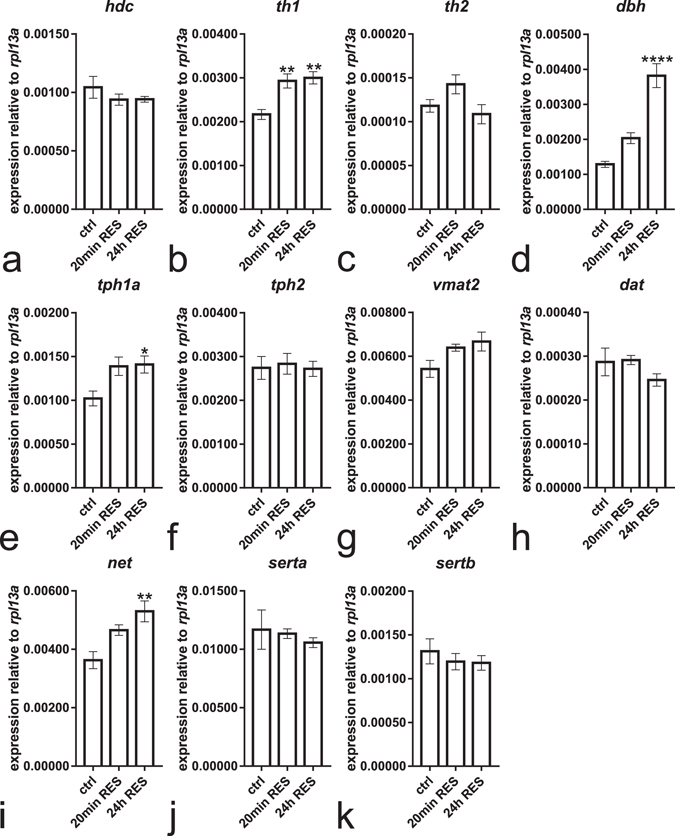
Analysis of mRNA expression levels 72 h after start of reserpine treatment in larvae treated for 20 min and 24 h. The rate-limiting enzyme in histamine synthesis, *hdc*, was not upregulated in the reserpine treated groups (**a**). The rate-limiting enzyme in dopamine synthesis, *th1*, was significantly upregulated in the reserpine treated groups (**b**) whereas *th2* showed no significant change in expression level (**c**). In the 24 h treated group, a significant increase was also seen in the transcript levels of *dbh* (**d**) and *tph1a* (**e**), the enzymes responsible for the synthesis of noradrenaline and serotonin, respectively. No change was seen in the expression of *tph2* (**f**). Out of the monoamine transporters, only the noradrenaline transporter *net* was significantly upregulated in the 24 h treated group, while *vmat2*, *dat*, *serta*, and *sertb* remained unchanged. *n* = 4 for controls and *n* = 5 for reserpine treated groups. Statistical analyses were done using 1-way ANOVA followed by Tukey’s multiple comparison test. Data presented as mean ± SEM. *p < 0.05, **p < 0.01 and ****p < 0.0001 according to the multiple comparison test, in comparison to the control group.

As we found that histamine was depleted from reserpinized fish, we asked whether such a loss of histamine would cause a compensatory increase in the transcription of genes important for histaminergic neurotransmission. Interestingly, no change in the mRNA for *histidine decarboxylase* (*hdc*), the rate-limiting enzyme for histamine biosynthesis, was seen at three days after reserpine treatment compared to controls (Fig. 6a). In contrast, the expression of mRNA for *tyrosine hydroxylase 1* (*th1*, F(2,11) = 9.515 *p* = 0.0040), a rate-limiting enzyme for dopamine biosynthesis, was significantly upregulated when compared with controls (Fig. 6b), while the expression of *tyrosine hydroxylase 2* (*th2*) was unchanged (Fig. 6c). In the 24 h treated group, we also observed a significant increase in the expression of *dopamine beta-hydroxylase* (*dbh*) and *tryptophan hydroxylase 1a* (*tph1a*), the enzymes responsible for the synthesis of noradrenaline and serotonin, respectively (Fig. 6d, F(2,11) = 29.98 *p* < 0.0001 and e, F(2,11) = 4.552 *p* = 0.0363). Out of the main monoamine transporters, only the *noradrenaline transporter* (*net*) was significantly upregulated in the 24 h treated group (Fig. 6i, F(2,11) = 8.178 *p* = 0.0067), while other monoamine transporters were unaffected (Fig. 6g–k).

### Histamine and other monoamine levels are decreased in reserpinized fish

**Figure 7:**
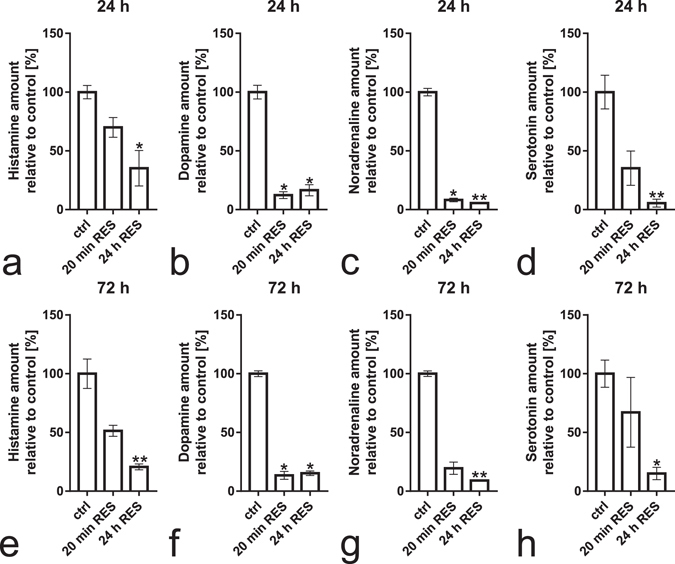
Monoamine levels measured by HPLC at 24 h and 72 h after the start of the reserpine treatment. At both time points, histamine levels were significantly decreased in comparison to controls in the 24 h treated group (**a**,**e**). The 20 min treated group follows a similar trend, however, this observation did not reach statistical significance. Dopamine levels were significantly decreased at both time points in both reserpine treated groups (**b**,**f**). Noradrenaline levels were decreased at both time points in the group treated with reserpine for 24 h (**c**,**g**). In the group treated with reserpine for 20 min, noradrenaline levels were significantly decreased at 24 h after treatment and followed a similar trend at 72 h after treatment, although the difference at this time point was not statistically significant (**c**,**g**, post-test p = 0.0771 in **g**). Serotonin levels were significantly decreased in the 24 h group at both time points (**d**,**h**). Data presented as mean ± SEM. n = 4–5 per group. Statistical analyses were done using Kruskal-Wallis test, followed by Dunn’s multiple comparison test. *p < 0.05, **p < 0.01 according to the multiple comparison test, in comparison to the control group.

At 24 h and 72 h after administration of reserpine, both reserpine treated groups showed decreased levels of histamine, although the effect reached statistical significance only for the group treated for 24 h (Fig. 7a, H(2) = 8.271 *p* = 0.0065 and e, H(2) = 12.5 *p* < 0.0001). Dopamine levels were significantly decreased in both reserpine treatment groups both 24 h and 72 h after treatment (Fig. 7b, H(2) = 8.28 *p* = 0.0062 and f, H(2) = 9.42 *p* = 0.0024). Noradrenaline levels were significantly decreased in both reserpine treatment groups at 24 h after treatment (Fig. 7c, H(2) = 10.22 *p* = 0.0006). At 72 h after treatment, noradrenaline levels were significantly decreased only in the group that received a 24 h reserpine treatment, with the group treated for 20 min showing non-significantly lowered levels (post-test p = 0.0771) (Fig. 7g, H(2) = 12.5 *p* < 0.0001). Serotonin levels were decreased in the group treated with reserpine for 24 h at both time points (Fig. 7d, H(2) = 10.21 *p* = 0.0007 and h, H(2) = 6.86 *p* = 0.0236).

## Discussion

We found that histamine immunoreactivity in the brain was strongly diminished by reserpine treatment both in neuronal cell bodies and, more importantly, their projections in key target areas in the dorsal telencephalon and habenula. This intraneuronal histamine represents the neurotransmitter pool of histamine, and thus its depletion should functionally impair histaminergic neurotransmission. Interestingly, the immunoreactivity in cell bodies was not uniformly diminished, with some cells retaining a clear cytoplasmic immunoreactivity and some cells showing a completely negative staining. Muroi *et al*. suggested the possibility of separate reserpine-sensitive and reserpine-resistant pools of histamine in neurons, with the reserpine-resistant pool being larger^15^. A relative sparing of immunoreactivity in some neurons could support this hypothesis to some extent, with the neurons showing a retained immunoreactivity being also more resistant to reserpine. Interestingly, the number of neurons preserved was similar in both treatment groups and all time points observed, with the number of cells showing clear histamine immunoreactivity decreased by about 40% following reserpine treatment. Further studies are needed in order to elucidate the reason for this heterogeneity. We found that all *hdc* mRNA positive neurons express *vmat2* mRNA. There is evidence of *vmat1* expression in the developing rat brain^20^. Thus, we also investigated the possibility that *vmat1* might be expressed in a subset of histaminergic neurons. However, we saw no expression of *vmat1* in the zebrafish brain at this stage using *in situ* hybridization. We therefore suggest that it is unlikely that the sparing of immunoreactivity in some histaminergic neurons would be explained by these neurons coexpressing another transporter. Our study presents strong data to support the hypothesis that histamine storage in neurons is VMAT2-dependent.

Our chromatographical data generally showed decreased levels of histamine, which is in agreement with the earlier chromatographic analyses of brain histamine after reserpine treatment in different species^13, 14, 21^. More recent studies have reported unaltered histamine levels, but even in these cases reserpine treatment significantly lowered the levels of histamine metabolites and inhibited pharmacologically induced increases in histamine^10, 15^. We also detected greatly decreased levels of other monoamines, which were lower than those found in VMAT2-heterozygous fish and of the same magnitude as those found in several other reserpine treated animal species^1, 22^. The chromatographic analysis of brain histamine in rodents is complicated by the presence of histamine in mast cells. The study by Maldonado & Maeyama accounted for this effect by using mutant rats lacking mast cells^10^. The zebrafish lacks mast cell histamine, showing immunoreactivity only in neurons^17^. An inherent weakness of chromatographic measurements is the inability to localize histamine to the actual neurons, and thus histamine levels in brain tissue samples might not accurately reflect the amount of histamine stored in neurons. This might to some degree explain the different results obtained in previous studies. In our study, the neural depletion was verified by histamine immunohistochemistry. As we measured the catecholamine and serotonin levels from whole larval homogenates, the observed decreases might also reflect changes in monoamine levels in other organs besides the brain. However, as *vmat2* in zebrafish is primarily expressed in the aminergic regions of the brain^23, 24^, it is likely that the effects on monoamines seen after vmat2 inhibition by reserpine would primarily reflect changes in the brain. However, as vmat2 has also been described to have functional roles in the peripheral nervous system in many species^25^, it is plausible that some of the effects observed reflect changes in the peripheral nervous system.

The increase in locomotion we observed during the short dark phase has been previously described by Burgess *et al*. as a form of dark adaptation^18^. The neural circuitry that initiates this behaviour is largely unknown, although Burgess *et al*. showed that dark-induced increases in locomotion were not dependent on the function of the Mauthner cells known to initiate rapid escape reactions in the zebrafish^18, 26^. We found that reserpine treatment significantly increased the magnitude of the dark-induced increase in locomotion at 24 h after administration of reserpine, which suggests that the circuitry regulating this response is negatively regulated by brain monoamines. In other animal models, reserpine has been shown to induce anxiety-like behaviour^27–29^, although this effect has not been seen in all studies^30^. The dark-induced short-lasting hyperactivity has been suggested to represent shelter seeking behaviour after nightfall or a reaction to find a way back to light after sudden onset of darkness^18, 31^. In nature, such a situation could be caused by an approaching predator that casts a shade on the fish. The increased magnitude of this response may therefore represent a form of increased anxiety. A recent study showed that VMAT2-heterozygous zebrafish have increased dark-avoidance and an anxiety-like phenotype in the novel tank test^22^. Recently, a group of reticulospinal (RS) neurons located in the nucleus of medial longitudinal fasciculus (nMLF) were identified as key modulators of locomotion velocity^32^. As this area also displays innervation by monoamine systems^33–35^, it is possible that the neural mechanism behind the increased locomotion during a dark cue in reserpine treated fish is due to decreased modulation of the nMLF due to acute monoamine depletion. It is known that dopamine negatively modulates the initiation of locomotion and movement in larval zebrafish^36^. A study on the role of noradrenaline in sleep found that noradrenaline-lacking *dbh* knock-out zebrafish display a decreased arousal threshold for tapping stimuli, implying that noradrenaline negatively regulates locomotor responses to sudden stimuli^37^. Further studies will be needed in order to discover the exact monoamine network in question.

Although the general locomotion during the sudden dark phase was increased in the treated animals, the dark-induced flash response observed during the first second of darkness was progressively weakened during the days following reserpine treatment, and the flash response was absent in repeated light-dark transitions. In a previous study by our group, we showed that this flash response is strongly histamine-dependent and mediated by histamine receptor H1, as knockdown of histamine synthesis and pharmacological inhibition of histamine receptor H1 abolish this response^19^. The weakened flash response observed could therefore be explained by the reserpine induced depletion of neural histamine. Our results also suggest that the underlying neural circuitry regulating the general increased locomotion following sudden darkness and the flash response during the first second of darkness is different. Ahrens *et al*. discovered that certain neurons in areas of the dorsal telencephalon and habenula respond to the fish being in either light or darkness^38^. The dorsal telencephalon and habenula are the main targets of histamine neurons and strongly express histamine receptors^19^, suggesting a histaminergic regulation of these light and dark responding neurons either directly or through local interneurons. In a recent study, serotonin has also been implicated to regulate dark avoidance behaviour in zebrafish larvae. Certain *tph2* expressing serotonergic neurons show high activity in darkness and inhibition of their function reduces dark avoidance behaviour in larvae^39^. However, our results would suggest that serotonin is not responsible for an increased locomotion in darkness, as serotonin levels were generally decreased in the reserpine treated larvae. The precise role of other monoamine systems than histamine in the regulation of the dark-induced flash response has yet to be determined.

After discovering that brain histamine levels are lowered by reserpine, we hypothesized that this might result in an upregulation of histidine decarboxylase (Hdc), the protein responsible for histamine synthesis. This idea is supported by earlier studies indicating that brain Hdc activity is increased in reserpinized mice^10^. We found, however, that *hdc* mRNA levels were completely unaltered in all reserpine treatment groups 72 h after the administration of reserpine. Interestingly, *tyrosine hydroxylase 1* (*th1*), *tryptophan hydroxylase 1a* (*tph1a*) and *dopamine beta-hydroxylase* (*dbh*), the enzymes encoding the rate-limiting steps in the biosynthesis of dopamine, serotonin and noradrenaline, respectively, were significantly upregulated, implying that monoamine depletion stimulates synthesis of the other monoamines at the transcriptional level. This suggests that the increased Hdc activity observed in reserpinized mice might be due to post-translational modification of the enzyme rather than activation of *hdc* transcription. Again, as whole larvae were used to obtain the mRNA samples, the changes in gene expression observed might reflect transcriptional changes in both the peripheral and central nervous system, as discussed previously in the context of the HPLC results.

Taken together, our results provide strong evidence that histaminergic neurotransmission is inhibited by treatment with reserpine, showing an almost total depletion of histamine fiber immunoreactivity following reserpine treatment. The reduction in the histamine-dependent dark-flash response suggests that this depletion is functionally relevant. We thus suggest that the storage of neuronal histamine and histaminergic neurotransmission is VMAT2 dependent, and that histamine storage and release in neurons works through a similar vesicular mechanism as observed for other monoamine neurotransmitters. Our results also implicate an inhibitory role of the monoaminergic network in the increased locomotion occurring during dark adaptation, a mechanism requiring further studies using fish strains deficient of specific monoamine receptors.

## Methods

### Experimental animals

Larvae of the wild-type Turku strain of zebrafish (*Danio rerio*) were used in all experiments. This strain has originally been maintained in the laboratory for over a decade and has been used in several earlier studies^19, 35^. The fish were maintained and bred according to Westerfield and as described earlier^40, 41^. 4–7 dpf (days post fertilization) larvae were used for the experiments. The larvae were killed mechanically after immersion in ice-cold water. The study was carried out in compliance with the ARRIVE guidelines. The animal project permit was obtained from the Animal Experiment Board of the Regional State Administrative Agency of Southern Finland. All experimental protocols were approved by it.

### Reserpine treatment

The larvae were raised in E3 medium on a Petri dish as described earlier^42^. At 4 dpf, the larvae were exposed to 40 mg/L of reserpine for 20 min or 24 h. The drug concentration and treatment of short duration were based on a previous study^43^. Since reserpine has not previously been used with zebrafish larvae, a longer, 24 h treatment was also used in the study (Fig. 1). Reserpine (Sigma-Aldrich 83580) was dissolved in E3 medium and the solution was agitated until no undissolved particles were visible. Larvae were assigned to the groups at random. At this stage, larvae cannot be identified from one another. All larvae used in each experiment came from the same breeding batch of fish and had a mixed parentage. After drug treatment, the larvae were washed three times with E3 and moved to a clean Petri dish. The control group was treated in the same way, but the treatment solution contained only E3. No increased mortality or developmental defects were seen after the treatment.

### Behavioural analysis

All behaviour trials were done using the Daniovision system (Noldus, Wageningen, The Netherlands). The larvae were transferred individually to a 48-well plate, each well prefilled with 1 ml 1xE3. The behavioural trial was conducted at 24 and 72 h after the start of reserpine treatment (Fig. 1). Different animals were used at each time point. The illuminance for the light phase was set at approximately 330 lux, in accordance with our previous work^19^. Before each trial, the larvae were habituated to the environment for 10 min, followed by a 15 min standard locomotion tracking with lights on. In order to evaluate the histamine-regulated dark-induced flash response described in our previous work^19^, the locomotion tracking was followed by 3 alternating lights on and lights off phases, with each phase lasting 2 min. All behavioural trials were done between 12:00 and 16:00.

### Histamine immunohistochemistry

Larvae were killed in ice-cold water and fixed overnight in freshly prepared 4% 1-ethyl-3-(3-dimethylaminopropyl)carbodiimide (EDAC) in 0.1 M sodium phosphate buffer (PB) pH 7.00 at +4 °C. After fixation, the larvae were washed briefly with 1xPBS and the brains were dissected under a stereo microscope. Immunohistochemistry was carried out as described earlier^40^. As primary antibody, a polyclonal rabbit anti-histamine antibody 19 C (RRID:AB_2314639, characterized previously, ref. 44) was used at a dilution of 1:10 000. This antibody does not show cross reactivity with L-histidine, histidine-containing peptides or other neurotransmitters^17, 35^. As secondary antibody, a commercial Alexa 488 conjugated monoclonal goat anti-rabbit antibody was used at a dilution of 1:1000 (RRID:AB_2576217, ThermoFisher A11034).

### RNA extraction and cDNA synthesis

For each RNA sample, 5 larvae were pooled in a 1.5 ml microcentrifuge tube. Excess liquid was removed, the tubes were flash-frozen in liquid nitrogen and stored at −80 °C until RNA isolation. RNA was isolated using the Qiagen RNeasy Mini kit (Qiagen, Hilden, Germany), following the manufacturer provided protocol for animal tissues, including a DNase I treatment to remove genomic DNA. 500–1000 ng of RNA was used for reverse transcription using the SuperScriptIII reverse transcriptase kit.

### Quantitative PCR

All qPCR analyses were performed using the LightCycler^®^ 480 system with the LightCycler® 480 SYBR Green I Master reaction mixture (Roche, Mannheim, Germany). A 1:10 dilution of cDNA was used as a reaction template, and the reaction mixture was prepared according to the manufacturer’s instructions. The following genes relevant to the function of the monoaminergic systems were analyzed: *histidine decarboxylase* (*hdc*), *tyrosine hydroxylase 1* (*th1*), *tyrosine hydroxylase 2* (*th2*), *tryptophan hydroxylase 1a* (*tph1a*), *tryptophan hydroxylase 2* (*tph2*), *dopamine beta-hydroxylase* (*dbh*), *dopamine transporter* (*dat*), *noradrenaline transporter* (*net*), *serotonin transporter a* (*serta*), *serotonin transporter b* (*sertb*) and *vesicular monoamine transporter 2* (*vmat2*). The quantification was done according to the comparative C(T) method^45^, using the gene *rpl13a* (*ribosomal protein L13a*) as an internal control. This gene has been described as a stable internal control in the zebrafish by previous studies^46, 47^. Primer sequences have been described earlier for *hdc*, *th1*, *th2*, *rpl13a* ^48^. Sequences of other primers were as follows: *tph1a*, 5′-CTGCCTGAGGAAAGCGAGAT-3′ and 5′-CATACATCAGCACGCGGTTC-3′; *tph2*, 5′-GGGCTGTGCAAACAAGATGG-3′ and 5′-CTCCTGGTAGCACGTGGTTT-3′; *dbh*, 5′-TGCAACCAGTCCACAGCGCA-3′ and 5′-GCTGTCCGCTCGCACCTCTG-3′; *serta*, 5′-ACAACCGATGGAACACTCCC-3′ and 5′-CAACACCTGCCGGACATAAA-3′; *sertb*, 5′-AGGAGACCAGCGTATGGGTA-3′ and 5′-GGGATTGTAGCTGGACAGGG-3′; *net*, 5′-TGATGGGCTTCAAACCAGGG-3′ and 5′-GTCAGTCGTCCGGAGGTAAC-3′; *dat*, 5′-CGTCACCAACGGTGGAATCTA-3′ and 5′-TGCCGATGGCCTCAATTAGTA-3′; *vmat2*, 5′-TGGAGCTCTGCAGCTTTTTGTGC-3′ and 5′-AACGCCGGCTCCAGCATAGC-3′.

### Histamine and monoamine measurement using high-performance liquid chromatography (HPLC)

For each sample, 15 zebrafish larvae were pooled and flash-frozen in liquid nitrogen. Samples were homogenized in approximately 10 volumes of 2% perchloric acid as described earlier (Eriksson *et al*. 1998). The HPLC analyses were done as described previously^17, 42^. The protein concentration of the samples was measured using the Pierce^©^ BCA Protein Assay Kit, and the HPLC data was normalized per total protein concentration of the sample.

### Cloning of a fragment of zebrafish vmat1

In order to create a *vmat1* riboprobe, a 735 bp fragment of the zebrafish *vmat1* mRNA (NCBI accession number XM_009304250.2) was amplified from cDNA from 7dpf wild-type untreated zebrafish larvae using the following primers: 5′-ACAACACCACCACTTACAACACC and 5′-GCACATCGTTTGCATCATCCAGATAG using the DyNAzyme II DNA polymerase kit (Thermo Scientific F-551). The PCR fragment was extracted from an 1% agarose gel using the MinElute Gel Extraction Kit (Qiagen, Hilden, Germany) and ligated into a pGEM®-T easy vector (Promega A1360, Fitchburg, WI, USA) and the resulting plasmid was transformed into DH5α bacteria. The insert was verified by sequencing. An antisense probe for ISH was synthesized using the DIG RNA labeling kit (Roche, Mannheim, Germany), as described earlier^48^.

### Fluorescent and chromogenic whole mount *in situ* hybridization

Larvae were fixed in 4% PFA (paraformaldehyde) in phosphate buffered saline (PBS), pH 7.4 overnight, followed by dissection of the brains. The dissected brains were stored in methanol at −20 °C until use. Fluorescent *in situ* hybridization was carried out according to Lauter *et al*. with minor modifications^49^. For detection of the labelled antisense probes, anti-DIG-POD and anti-FLU-POD Fab-fragments (RRID: AB_514500 and AB_840257, Roche, Mannheim, Germany) were used at a concentration of 1:2000. Fluorescent detection was done using tyramide signal amplification for 15 min with a 1:250 dilution of TAMRA (5-(and-6)-Carboxytetramethylrhodamine) (ThermoFisher C1171) or DY-647P1 (Dyomics) conjugated tyramide synthesized as described by Lauter^49^. Fluorescein labelled *hdc* and digoxigenin labelled *vmat2* riboprobes were used and synthesized as described earlier^48^.

Chromogenic *in situ* hybridization was done according to the Thisse lab protocol^50^. Digoxigenin labelled *vmat1* and *vmat2* riboprobes were used for hybridization. The probes were detected using anti-DIG-AP Fab-fragments (RRID: AB_514497, Roche, Mannheim, Germany) and the chromogenic color reaction was developed using NBT (nitro blue tetrazolium chloride) and BCIP (5-bromo-4-chloro-3-indolyl phosphate).

### Microscopy and imaging

All fluorescent images were acquired using a Leica SP2 AOBS confocal microscope (Leica, Wetzlar, Germany) using a 488 nm argon laser, a 561 nm diode laser and a 633 nm helium/neon laser for fluorophore excitation. The emission ranges were selected at 500–550 nm, 570–622 nm and 650–750 nm, respectively. Cross-talk between the channels was excluded by sequential scanning and controlling that no bleed-through occurred using single fluorophore samples. The whole brain was visualized for cell counting using a HC PL APO 20x/0.70 CS objective, and close-up images were taken using a HC PL APO 40x/0.75 CS objective. Image stacks were analyzed and cells counted using Fiji software^51^ and cell counting was performed blinded to the sample group.

Chromogenic samples were imaged using a Leica DM IRB inverted microscope. All samples were imaged using the same exposure settings. Scalebars were added to the images using Fiji software.

### Statistical analyses

Numerical data was analyzed using one-way ANOVA followed by Tukey’s post-hoc test in Graph Pad Prism 7 software (Graph Pad Software, San Diego, CA, USA). The time-nested behaviour data was analyzed using two-way ANOVA followed by Tukey’s post-hoc test. In situations where variance between groups was significantly different as assessed by Bartlett’s test, the non-parametric Kruskal-Wallis test was used instead of one-way ANOVA. Kruskal-Wallis test and Dunn’s post hoc test with Benjamini-Hochberg correction^52^ of multiple comparisons were computed using R version 3.3.3 (R Development Core Team, Vienna, Austria). Sample sizes used were estimated in accordance with our previous studies^19, 48^, and each *n* represents an independent experimental unit. The confidence interval was set at 95%.

### Data availability statement

The datasets generated during and/or analysed during the current study are available from the corresponding author on reasonable request.
